# Identification of miRNAs induced by low-dose methylmercury exposure and their roles in inflammatory responses using human aortic endothelial cells

**DOI:** 10.1265/ehpm.25-00292

**Published:** 2025-11-28

**Authors:** Rika Matsuyama, Athira Nandakumar, Munekazu Yamakuchi, Saekhol Bakri, Shiroh Tanoue, Mayumi Tsuji, Megumi Yamamoto, Teruto Hashiguchi, Chihaya Koriyama

**Affiliations:** 1 Department of Epidemiology and Preventive Medicine, Kagoshima University Graduate School of Medical and Dental Sciences, Kagoshima, Japan; 2 Department of Laboratory and Vascular Medicine, Kagoshima University Graduate School of Medical and Dental Sciences, Kagoshima, Japan; 3 Department of Public Health, Faculty of Medicine, Universitas Diponegoro, Semarang, Central Java, Indonesia; 4 Department of Environment Health, University of Occupational and Environmental Health, Kitakyushu, Japan; 5 Department of Environment and Public Health, National Institute for Minamata Disease, Minamata, Kumamoto, Japan

**Keywords:** Cyclooxygenase-2, Human aortic endothelial cells, Inflammation, Methylmercury, MicroRNAs

## Abstract

**Background:**

Exposure to methylmercury (MeHg) is predominantly attributed to consumption of marine products. However, the general population is exposed to low MeHg levels, which can induce chronic inflammation. Although some MeHg-related microRNAs (miRNAs) have been reported, their functions remain elusive. The objective of this study was to identify the miRNAs induced by low-level MeHg exposure in a human endothelial cell line (HAECs). This study aimed to determine the specific miRNAs induced by low-level MeHg exposure using a HAECs as a potential novel and sensitive biomarker. The roles of miRNAs in inflammatory processes have been examined.

**Methods:**

Using HAECs, a miRNA microarray assay was performed to identify miRNAs with altered expression upon exposure to a non-cytotoxic MeHg level (0.1 and 1.5 µM). The expression patterns of interleukin-6 and -8, cyclooxygenase 2 (COX-2), RelB, and prostaglandin E_2_ (PGE_2_) were examined after transfection of the identified miRNAs with mimics/inhibitors.

**Results:**

Although the microarray assay identified six MeHg-specific miRNAs, miR-3613-5p, upregulated by 0.1 and 1.5 µM MeHg exposures, demonstrated the best reproducibility in HAECs. Transfection with the miR-3613-5p mimic enhanced the MeHg-induced inflammatory responses, including PGE_2_ and COX-2 protein levels, whereas the miR-3613-5p inhibitor suppressed these inflammatory responses.

**Conclusion:**

This study observed that miR-3613-5p is induced by low-dose MeHg exposure, plays a crucial role in the inflammatory process, and could serve as a novel and sensitive biomarker for low-level MeHg exposure.

**Supplementary information:**

The online version contains supplementary material available at https://doi.org/10.1265/ehpm.25-00292.

## Introduction

Methylmercury (MeHg) is the most toxic of the three main forms of mercury—metallic Hg, inorganic Hg, and MeHg—owing to its high bioavailability and neurotoxic properties [1]. MeHg exposure levels in the general population are low, but the health effects of low-level exposure remain unclear, and research on potential biomarkers for non-cytotoxic MeHg exposure is limited.

Inflammation is a key indicator of tissue damage caused by both MeHg and inorganic Hg. Interleukin (IL)-6 and IL-8 have been reported at different doses of MeHg, including non-cytotoxic levels [2–4]. Nuclear factor-kappa B (NF-κB) and cyclooxygenase 2 (COX-2) play essential roles in MeHg-induced inflammatory pathways [4, 5]. MeHg induces COX-2 expression in vascular endothelial cells by inhibiting protein tyrosine phosphatase 1 B activity via the prostaglandins I_2_ and E_2_ (PGI_2_/PGE_2_)-activated cAMP pathway [5]. PGE_2_, released by COX-2, triggers various physiological and inflammatory responses [6].

MicroRNAs (miRNAs) regulate gene expression by interacting with messenger RNA (mRNA) and influencing its stability and translation. Circulating miRNAs in the blood are remarkably stable due to their protection from RNase degradation, rendering them useful biomarkers for *in vivo* responses [7]. As exposure to inorganic Hg and MeHg influences miRNA expression, miRNAs may be biomarkers of Hg exposure [8]. One study reported an increase in NF-κB activity and COX-2 expression by miRNAs induced by high-level HgCl_2_ exposure [9]. However, studies on miRNAs as potential biomarkers for predicting the health effects of low-level MeHg exposure are limited.

Recent studies have identified blood inflammatory reactions and reactive oxygen species (ROS) as risk factors for atherosclerosis, alongside traditional factors like aging, smoking, and diabetes. Moreover, a suspected connection was observed with both inorganic and organic mercury exposure [10, 11]. Furthermore, ROS activates NF-κB and activator protein 1, which regulate the pro-inflammatory genes expression [12]. Many miRNAs associated with inflammatory reactions, endothelial nitric oxide synthase phosphorylation, and nitric oxide production have been identified [13]. A significant proportion of miRNAs in the blood originates from vascular endothelial cells, underscoring their endothelial origin [14].

Thus, we aimed to explore specific miRNAs as novel and sensitive biomarkers associated with low-dose MeHg exposure and determine their functions in human aortic endothelial cells.

## Materials and methods

### Cell culture

Human aortic endothelial cells (HAECs; Lonza Walkersville, MD, USA) were cultured at 37 °C in a humidified incubator with 5% CO_2_ using endothelial cell growth basal medium and EBM2 medium supplemented with growth factors (CC-3162; Lonza Walkersville, MD, USA). Upon reaching 70–80% confluency, the cells were washed with 10 mL of PBS and treated with 1.5 mL of 0.25% Trypsin-EDTA for 5 min, followed by inactivation using EBM2 medium containing 10% fetal bovine serum (FBS; Thermo Fisher Scientific, Waltham, MA, USA).

### MeHg preparation

MeHgCl (10 mM; purity >95%; #26803-96 Kanto kagaku, Tokyo, Japan) was dissolved in Dulbecco’s PBS (Sigma-Aldrich) containing l-cysteine (Hg:Cys = 1:1) and stored at −80 °C until use. The prepared stock solution was diluted with culture medium immediately before use.

### Cytotoxicity assay

Cells were seeded in a 96-well plate at a density of 1 × 10^5^ cells/mL and cultured for 24 h. After an additional 24-h exposure to MeHg at varying concentrations (0–10 µM), cell viability was assessed using the WST-8 Cell Counting Kit (Wako Pure Chemical Industries, Osaka, Japan). Hank’s balanced salt solution (100 µL) and WST-8 solution (10 µL) were added, followed by incubation at 37 °C with 5% CO_2_. Color development was quantified at 450 nm within 1–2 h using a microplate spectrophotometer (TriStar LB941; Berthold Technologies, Germany).

### mRNA extraction and real-time PCR assay for inflammatory responses

mRNA extraction and real-time PCR were performed as described previously [5]. Briefly, after extracting total RNA from HAECs using an RNeasy Plus Mini kit (Qiagen, MD, USA), complementary DNA (cDNA) was synthesized from 600 ng of total RNA using a QuantiTect Reverse Transcription kit (Qiagen, MD, USA). Expression levels of IL-6, IL-8, COX-2, and RelB (a key component of NF-kB) mRNAs were quantified using the LightCycler system (Roche Diagnostics, Switzerland) with LightCycler FastStart DNA MasterPLUS SYBR Green I (Roche Diagnostics, Switzerland). Primer design and synthesis were based on information from the NCBI and Primer3 software (Greiner Bio-One, Japan), targeting gene amplicons of 80,300 bp (Sigma-Aldrich, Japan). The primers used are listed in Supplementary Fig. 1. DNA polymerase was activated by initial denaturation at 95 °C for 10 min, followed by 45 amplification cycles comprising denaturation at 95 °C for 10 s, annealing at 60 °C for 10 s, and extension at 72 °C for 15 s. All assays were performed in triplicate.

### miRNA preparation and reverse transcription-quantitative PCR

In brief, 700 µL of Qiazol Lysis Reagent (Qiagen, MD, USA) was added to HAECs exposed to MeHg or control for 24 h. Then, miRNAs were extracted using the miRNeasy Mini kit (Qiagen, MD, USA) and eluted with 50 µL of RNase-free water. All procedures were performed in duplicates and repeated four times.

We used the TaqMan MicroRNA Reverse Transcription Kit (Applied Biosystems) to synthesize our samples. We performed qPCR. The mixtures were incubated at 16 °C for 30 min, 42 °C for 30 min, and 85 °C for 5 min in 40 amplification cycles. To calculate miRNA expression levels, U6 (Thermo Fisher Scientific) was used as a reference.

### miRNA microarray analysis

HAECs were seeded at a density of 1 × 10^5^ cells/well in a 6-well plate and cultured for 24 h. MeHg (0.1 and 1.5 µM) was added and cells were harvested 24 h later with a control without MeHg. Microarray analysis was performed using three samples corresponding to 0, 0.1, and 1.5 µM MeHg exposure. Quality of total RNA was checked with the Agilent 4150 TapeStation, labeled with the FlashTag™ Biotin HSR RNA Labeling Kit (Thermo Fisher Scientific), and processed for hybridization, washing, and scanning per the manufacturer’s protocols. Data were analyzed using Transcriptome Analysis Console v4.0. Microarray analysis was performed at Cell Innovator Corporation (Fukuoka, Japan).

### Transfection of HAEC with miRNA mimic and inhibitor

Based on miRNA array results and subsequent experiments, miR-3613-5p was identified as a candidate MeHg-specific miRNA. The miR-3613-5p mimic and inhibitors (Thermo Fisher Scientific) were incubated with Lipofectamine RNAiMAX (Thermo Fisher Scientific). MirVana miRNA mimic (4464058) and mirVana miRNA inhibitor (4464076) (Thermo Fisher Scientific) were used as negative controls. Transfected HAECs were cultured for an additional 48 h, followed by exposure to 0.1 and 1.5 µM of MeHg for 3 h.

### Protein expression analysis

In supernatants of HAECs transfected with miR-3613-5p-mimic/inhibitor, the PGE_2_ concentration was determined using the PGE_2_ highly sensitive ELISA kit (Arbor Assays, Ann Arbor, MI, USA).

Transfected HAECs were exposed to 0.1 or 1.5 µM MeHg for 6 h, followed by sodium dodecyl sulfate-polyacrylamide gel electrophoresis for western blotting. The proteins were transferred to nitrocellulose membranes (Bio-Rad, USA) at 450 mA for 30 min, blocked in PBS containing 1% bovine serum albumin (BSA; Sigma-Aldrich, MO, USA), and incubated with 50 ng monoclonal antibody against COX-2 (MP Biomedicals, Santa Ana, CA, USA). After washing with PBS containing 0.05% Tween 20, the membranes were incubated with a peroxidase-labeled anti-mouse antibody (Agilent Technologies, Santa Clara, CA, USA) in PBS containing 3% BSA. Membranes were washed and subjected to chemiluminescent band detection using ECL Western blotting detection reagent (Merck KGaA, Darmstadt, Germany) and MultiImage II (ProteinSimple, Japan). β-actin was used as an internal control. The primary antibodies used were mouse IgG and horseradish peroxidase-linked sheep whole antibodies (Amersham ECL, catalog no. NA931). All assays were performed in triplicate. Band intensities were quantified using the ImageJ software and normalized to β-actin levels. The relative expression levels (sample/control ratios) were calculated from three independent experiments.

### Statistical analysis

The Shapiro-Wilk test was used to analyze data normality. Because cell culture conditions in each experiment could potentially influence the results of miRNA mimic/inhibitor transfection, control and mimic/inhibitor-treated cells were treated as paired samples. For the expression levels of inflammatory cytokines, since they followed a normal distribution, a paired t-test was performed. For the comparison between the control group without MeHg exposure and the exposed group, we treated them as two independent groups and performed the Wilcoxon rank-sum test. The effects of multiple comparisons were adjusted for using Holm’s method. Statistical significance was set at P < 0.05.

## Results

### Cytotoxicity assay

At MeHg concentrations of 0.1, 1.5, and 2 µM, HAECs demonstrated approximately 100%, 97%, and 80% viability, respectively. Accordingly, in subsequent experiments, 1.5 µM MeHg was selected as the maximum non-cytotoxic concentration (Supplementary Fig. 2).

### Identified miRNAs affected by MeHg exposure in HAECs

Based on the miRNA microarray analysis of 6,599 miRNAs, we identified six miRNAs that demonstrated a more than two-fold change in upregulation or downregulation upon MeHg exposure compared to the control (MeHg-untreated cells) (Table 1).

**Table 1:** List of miRNAs affected by MeHg exposure in human aortic endothelial cells*

**MeHg** **concentration**	**miRNA**	**Effect**	**Fold** **Change**
0.1 µM	miR-3613-5p	up	9.917
	miR-500a-3p	up	2.027
	miR-139-5p	down	−2.5
	miR-140-3p	down	−3.98
	miR-4284	down	−3.01

1.5 µM	miR-3613-5p	up	9.917
	miR-138-1-3p	up	3.53
	miR-139-5p	down	−3.58
	miR-4284	down	−2.58

*HAEC cells were treated with 0 µM (control), 0.1 µM, or 1.5 µM MeHg for 24 h. miRNAs identified by miRNA microarray assay, showing more than two-fold changes following MeHg exposure compared with the control.

### Reproducibility of expression of miRNAs identified by the microarray assay

**Fig. 1:**
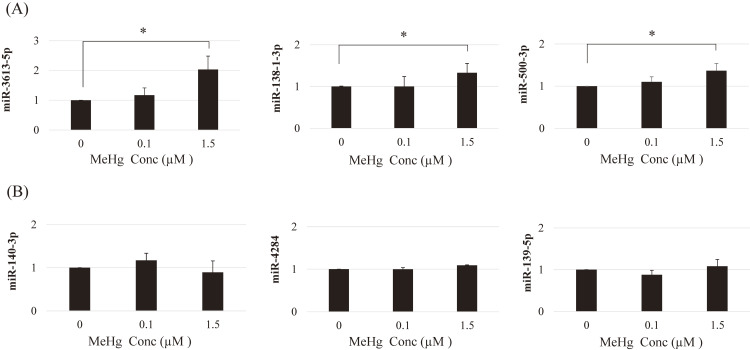
Expression of MeHg-induced miRNAs identified by microarray assays HAECs were incubated with MeHg (0.1 µM and 1.5 µM) for 24 h. Expression patterns of miR-3613-5p, miR-138-1-3p, and miR-500-3p were upregulated in the miRNA microarray assays (A). Expression patterns of miR-140-3p, miR-4284, and miR-139-5p were downregulated in miRNA microarray assays (B). Mean values and standard errors (error bars) from four independent experiments are shown (n = 8 for each group). The miRNA expression levels in the MeHg-exposed cells (0.1 µM or 1.5 µM) were compared with those in the control (MeHg: 0 µM) for each miRNA. *Statistically significant (adjusted p < 0.05)

We determined the expression patterns of the six miRNAs identified in HAECs exposed to 0.1 and 1.5 µM MeHg (Fig. 1). Expression of miR-3613-5p was significantly upregulated at 1.5 µM MeHg but not at 0.1 µM (Fig. 1A). Although miR-500-3p was upregulated at 0.1 µM MeHg only in the microarray assay, it showed dose-dependently upregulated expression in PCR results. Conversely, none of the miRNAs that were down-regulated in the microarray assay showed reduced expression (Fig. 1B). Only the miR-3613-5p and miR-138-1-3p expression levels were consistent with those observed in the miRNA microarray assays (Table 1). Due to the high reproducibility of miR-3613-5p expression following MeHg exposure, further experiments were performed using miR-3613-5p as a candidate miRNA.

### Assessment of miR-3613-5p involvement in MeHg-induced inflammatory responses

**Fig. 2:**
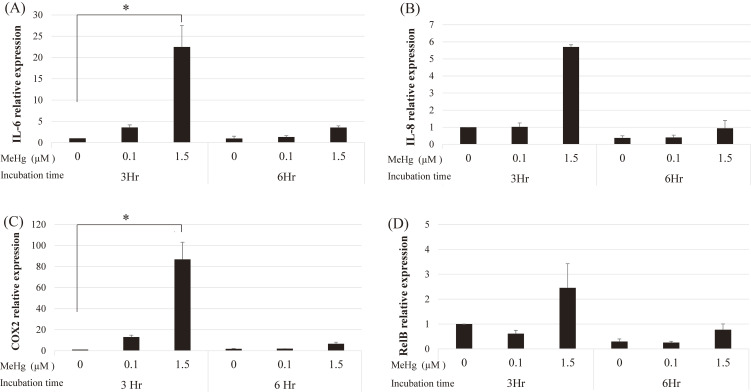
Inflammatory responses induced by MeHg exposure are reflected in the mRNA level in HAECs HAEC cells were treated with 0 µM (control), 0.1 µM, or 1.5 µM MeHg for 3 and 6 h. Considering the expression of β-actin, relative IL-6 (A), IL-8 (B), COX-2 (C), and RelB (D) expressions in HAECs. The mean values and standard errors (error bars) were obtained from three independent experiments (n = 3 for each group). The relative cytokine expression levels in the MeHg-exposed cells (0.1 µM or 1.5 µM) were compared with those in the control by cytokine and incubation time. *Statistically significant (adjusted p < 0.05)

In HAECs, exposure to 1.5 µM MeHg for 3 h significantly increased responses of IL-6 and COX-2 (Fig. 2).

**Fig. 3:**
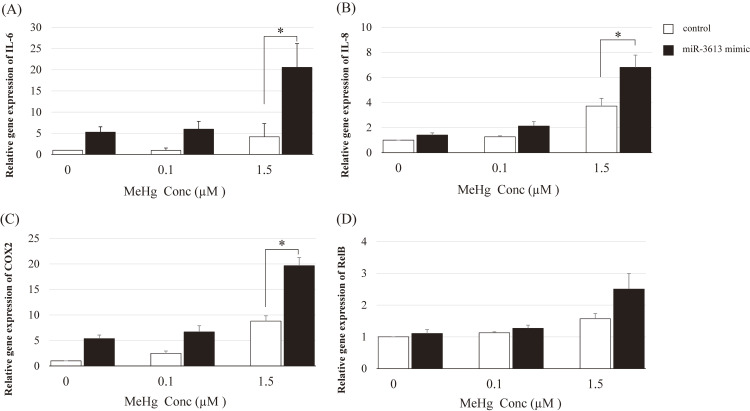
MeHg-induced inflammatory responses at the mRNA level after transfection with miR-3613-5p mimic HAEC cells were treated with 0 µM, 0.1 µM, or 1.5 µM MeHg for 3 h. Relative expression of MeHg-induced IL-6 (A), IL-8 (B), COX-2 (C), and RelB (D) in HAECs transfected with the miR-3613-5p mimic. Control: Oligonucleotides (double-stranded RNA). The mean values and standard errors (error bars) were obtained from four independent experiments (n = 4 for each group). The relative cytokine expression levels in cells treated with the miR-3613-5p mimic were compared to those in the control (without the miR-3613-5p mimic) using MeHg levels. *Statistically significant (adjusted p < 0.05)

**Fig. 4:**
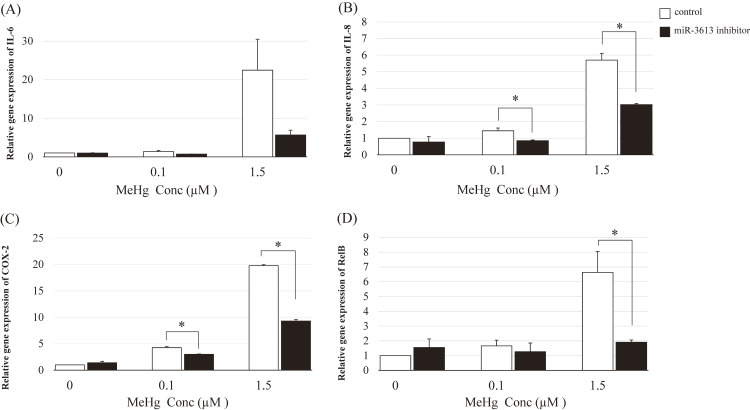
MeHg-induced inflammatory responses at the mRNA level after transfection with the miR-3613-5p inhibitor HAEC cells were treated with 0 µM (control), 0.1 µM, or 1.5 µM MeHg for 3 h. Relative expression of MeHg-induced IL-6 (A), IL-8 (B), COX-2 (C), and RelB (D) in HAECs treated with the miR-3613-5p inhibitor. Control: oligonucleotide (single-stranded RNA). The mean values and standard errors (error bars) were obtained from four independent experiments (n = 4 for each group). The relative cytokine expression levels in the cells treated with the miR-3613-5p inhibitor were compared with those in the control (without the miR-3613-5p inhibitor) by MeHg level. *Statistically significant (adjusted p < 0.05)

Transfection with the miR-3613-5p mimic enhanced MeHg-induced inflammatory responses, particularly the expression of IL-6, IL-8, and COX-2 (Fig. 3A, B, and C, respectively). The miR-3613-5p mimic did not significantly enhance RelB expression even at 1.5 µM MeHg (Fig. 3D). Conversely, the miR-3613-5p inhibitor significantly suppressed the MeHg-induced expression of IL-8, COX-2, and RelB, especially at 1.5 µM MeHg level (Fig. 4).

### Expression of PGE_2_ and COX-2 following MeHg exposure after transfection with miR-3613-5p mimic/inhibitor

**Fig. 5:**
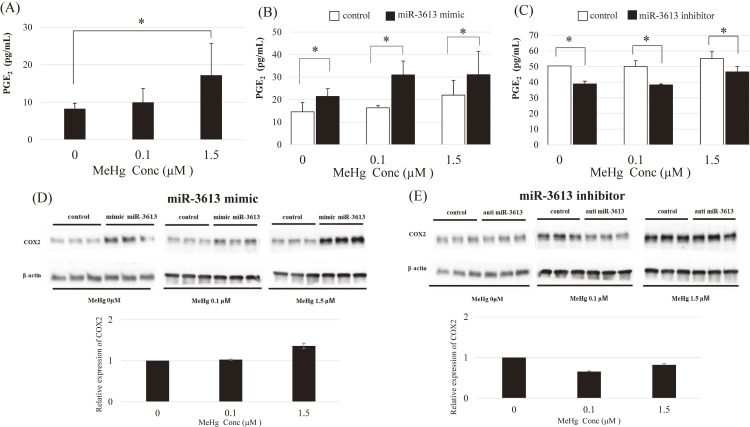
MeHg-induced PGE_2_ and COX-2 expressions at the protein level after transfection with miR-3613-5p mimic/inhibitor HAEC cells were treated with 0 µM (control), 0.1 µM, or 1.5 µM MeHg for 3 h. MeHg dose-dependent PGE_2_ expression (A). MeHg enhanced PGE_2_ expression in HAECs transfected with the miR-3613-5p mimic (B). miR-3613-5p inhibitor suppresses MeHg-induced PGE_2_ expression in HAECs (C). MeHg enhances COX-2 expression in HAECs transfected with the miR-3613-5p mimic (D). miR-3613-5p inhibitor suppresses MeHg-induced COX-2 expression in HAECs (E). Control: Oligonucleotides (double- and single-stranded RNAs for the mimic and inhibitor, respectively). Mean values and standard errors (error bars) were obtained from triplicate experiments (n = 3). *Statistically significant (adjusted p < 0.05)

ELISA and western blotting were performed to determine the expression levels of PGE_2_ and COX-2 proteins. The production of PGE_2_ increased with MeHg exposure (Fig. 5A). Overexpression of miR-3613-5p, via transfection with its mimic, enhanced MeHg-induced PGE_2_ production (Fig. 5B). Transfection with a miR3613-5p inhibitor suppressed PGE_2_ protein production (Fig. 5C). Similarly, the miR-3613-5p mimic enhanced MeHg-induced COX-2 expression (Fig. 5D). In contrast, miR-3613-5p inhibitors suppressed this expression (Fig. 5E), although the suppression was not as pronounced as that observed for mRNA.

## Discussion

Using microarray analysis, we identified six miRNAs with altered expression following low MeHg exposure. Further mRNA and protein experiments using an miR-3613-5p mimic and inhibitor revealed that miR-3613-5p might be the key miRNA involved in the regulation of inflammatory responses induced by low MeHg levels. Therefore, this miRNA is considered a novel and highly sensitive candidate biomarker for low MeHg exposure.

Although previous studies examined the association between Hg exposure and miRNA expression, most focused on high-level Hg exposure, assuming occupational exposure [8, 15]. Upregulated miR-92a and miR-486 expressions were reported in workers exposed to high Hg levels [15], and these miRNAs induced activation of NF-κB and COX-2 in human umbilical vein endothelial cells and human embryonic kidney cells following exposure to high levels of inorganic Hg (5 and 10 µM) [9]. In this study, low MeHg levels did not affect the expression of these miRNAs according to the microarray results.

The involvement of miR-3613-5p in inflammatory response has been previously suggested. Bakhashab et al. [16] reported a crucial role of miR-3613-5p in mast cell differentiation and functional regulation mediated by IL-33. miR-3613-5p targets the transcription factors MITF and PPARGC1A, which influence mast cell survival and inflammatory responses, thereby contributing to inflammation regulation. Furthermore, miR-3613-5p may be involved in multiple signaling pathways in patients with mesial temporal lobe epilepsy, including the PI3K/Akt pathway, which plays a critical role in cellular survival responses to oxidative stress and promotes the expression of antioxidant enzymes [17]. HgCl_2_ or MeHg exposure is induces oxidative stress and activates cellular antioxidant responses via the PI3K/Akt pathway [18]. These findings suggested that miR-3613-5p plays a crucial role in MeHg-induced inflammation. Elucidating the molecular mechanisms by which miR-3613-5p regulates the expression of these inflammatory cytokines will lead to the development of new treatments and facilitate the modulation of inflammatory responses induced by MeHg.

This is the first report examining the inflammatory response to MeHg exposure using HAECs. Exposure of HAECs to low levels of MeHg-induced inflammatory responses is associated with MeHg. MeHg is demethylated from inorganic mercury. Long-term exposure to low HgCl2 levels can trigger the MAPK signaling pathway in vascular smooth muscle cells. This leads to the activation of inflammatory proteins, including nicotinamide adenine dinucleotide phosphate oxidase and COX-2 [19], resulting in endothelial dysfunction. Accordingly, chronic Hg exposure may induce vascular diseases.

We observed an increase in RelB expression upon exposure to low MeHg concentrations. Two known signaling pathways are associated with the activation of inactive NF-κB: the classical (canonical) and non-canonical pathways. RelB is a key component of NF-κB in non-canonical pathways. In this pathway, when a ligand binds to the receptor and activates it, NF-κB-inducing kinase and the IKK complex are sequentially activated, and through limited degradation, the p100/RelB complex becomes p52/RelB, which migrates to the nucleus and induces the expression of target genes [20]. MeHg induces inflammatory reactions via canonical pathways and RelA expression in U937 macrophages [3]. However, in the current study, MeHg exposure did not induce significant RelA expression in HAECs (data not shown). Whether this difference was due to differences in the cells used in the experiments remains unclear. The inflammatory reactions induced by MeHg exposure may involve both pathways. Further investigations are necessary to explore the detailed mechanisms underlying the inflammatory response to MeHg exposure.

In this study, the IL-6, IL-8, and COX-2 expression levels in cells treated with the miR-3613-5p inhibitor control were higher than those in cells treated with the miR-3613-5p mimic control. These observations may be due to the immunostimulatory effect of single-stranded RNA in the inhibitor control, which activates TLR7 signaling and enhances NF-κB-mediated inflammatory responses, whereas in the miR-3613-5p mimic, double-stranded RNA activates TLR3, which tends to induce type I interferon responses rather than pro-inflammatory cytokines [21, 22].

Collectively, this study confirmed that exposure to low MeHg levels can induce inflammatory responses in HAECs and that miR-3613-5p may be a key regulator of MeHg-induced inflammation. These findings suggest that miR-3613-5p could serve as a novel and sensitive biomarker for low-level MeHg exposure and provide novel insights into the molecular mechanisms underlying MeHg-induced inflammation.

## Abbreviations

MeHg: methylmercury
IL: interleukin
NF-κB: nuclear factor-kappa B
COX-2: cyclooxygenase 2
PGI_2_ and PGE_2_: prostaglandins I_2_ and E_2_
ROS: reactive oxygen species
miRNA: microRNA
mRNA: messenger RNA
HAECs: human aortic endothelial cells

## Supplementary information

**Additional file 1:** **Supplementary Fig. 1.** Primers for real-time quantitative RT-PCR.

**Additional file 2:** **Supplementary Fig. 2** Cytotoxicity of HAEC exposed to MeHg.

## Declarations

### Availability of data

Data available on request from authors.
