# Supplementary material for: Identification of miRNAs induced by low-dose methylmercury exposure and their roles in inflammatory responses using human aortic endothelial cells
Source: Environ Health Prev Med. 2025 Nov 28;30:93. doi: 10.1265/ehpm.25-00292 (PMC12678024; doi:10.1265/ehpm.25-00292)
Supplement: Supplementary file 1 — Additional file 1: Supplementary Fig. 1. Primers for real-time quantitative RT-PCR. [file ehpm-30-093-s001.pdf]

**Gene**	**Forward Primer**	**Reverse Primer**
β-actin	5'-GACC GTG CTG CTG ACC-3'	5'-CCA GAG GCG TAC AGG GAT AGC-3'
IL-6	5'-GAA CTC CTT CTC CAC AAG CG-3'	5'-TTT TCT GCC AGT GCC TCT TT-3'
IL-8	5'-GAC ATA CTC CAA ACC TTT CC-3'	5'-CTT CTC CAC AAC CCT CTG-3'
RelB	5'-TCC CAA CCA GGA TGT CTA GC-3'	5'-AGC CAT GTC CCT TTT CCT CT-3'
COX-2	5'-GGT CTG GTG CCT GGT CTG -3'	5'-ACA TAA TCT TCA ATC ACA ATC TTA ATA GTC-3'
