# Supplementary material for: Identification of miRNAs induced by low-dose methylmercury exposure and their roles in inflammatory responses using human aortic endothelial cells
Source: Environ Health Prev Med. 2025 Nov 28;30:93. doi: 10.1265/ehpm.25-00292 (PMC12678024; doi:10.1265/ehpm.25-00292)
Supplement: Supplementary file 2 — Additional file 2: Supplementary Fig. 2 Cytotoxicity of HAEC exposed to MeHg. [file ehpm-30-093-s002.pdf]

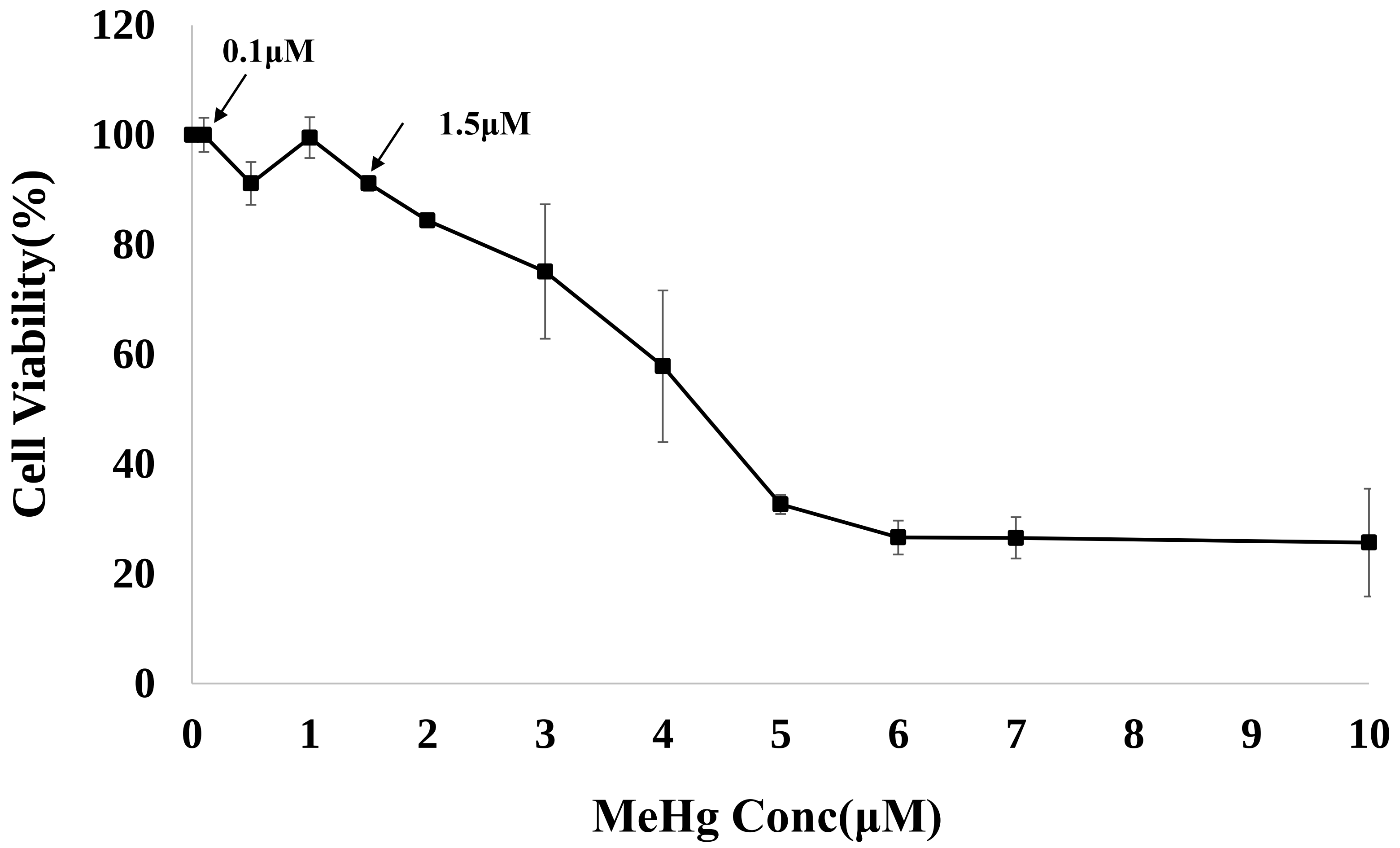

After HAECs were incubated with different MeHg concentrations (0–10 μM) for 24 h, cytotoxicity was determined. Cell viability was calculated using HAECs without MeHg treatment as a reference. The mean values and standard errors (error bars) of cell viability at different MeHg exposure levels are shown.
